# Flavivirus-Induced ER Stress and Unfolded Protein Response: A Central Hub Linking Lipid Droplet Remodeling and Viral Replication

**DOI:** 10.3390/v18050493

**Published:** 2026-04-23

**Authors:** Imaan Muhammad, Kaci Craft, Shaokai Pei, Ruth Cruz-Cosme, Qiyi Tang

**Affiliations:** Department of Microbiology, Howard University College of Medicine, Washington, DC 20059, USA; imaan.muhammad@bison.howard.edu (I.M.); kaci.craft@bison.howard.edu (K.C.); shaokai.pei1@bison.howard.edu (S.P.); ruth.cruzcosme@howard.edu (R.C.-C.)

**Keywords:** flavivirus, ER stress, unfolded protein response, lipid droplet, viral replication, Zika virus, protein kinase RNA-like ER kinase (PERK), inositol-requiring enzyme 1 (IRE1), activating transcription factor 6 (ATF6)

## Abstract

Endoplasmic reticulum (ER) stress and the unfolded protein response (UPR) represent fundamental cellular adaptive mechanisms that maintain protein homeostasis and metabolic balance. Many RNA viruses, particularly flaviviruses such as dengue virus (DENV), Zika virus (ZIKV), West Nile virus (WNV), yellow fever virus (YFV), and Japanese encephalitis virus (JEV), extensively remodel the ER to establish replication compartments and assemble progeny virions. This massive reorganization disrupts ER homeostasis, leading to UPR activation. Emerging evidence reveals that flaviviruses not only trigger but also manipulate the three UPR branches—PERK, IRE1, and ATF6—to optimize viral translation, replication, and egress. In parallel, flavivirus infection profoundly alters host lipid metabolism and promotes dynamic changes in lipid droplets (LDs), key organelles that mediate lipid storage and serve as scaffolds for viral replication and assembly. The UPR intimately connects to LD biogenesis through transcriptional and translational programs mediated by XBP1, ATF4, and ATF6, thereby coupling ER stress responses to lipid remodeling and energy homeostasis. This intricate crosstalk between UPR and LDs creates a metabolic and structural niche favorable for viral replication but detrimental to host cell integrity. This review provides a comprehensive analysis of the molecular mechanisms by which flaviviruses exploit ER stress and the UPR to reprogram lipid metabolism and LD dynamics. We highlight the dual role of UPR signaling in promoting adaptive lipid synthesis and initiating cell death under prolonged stress, discuss recent insights into ER–LD interactions during flavivirus infection, and explore therapeutic opportunities targeting UPR–lipid metabolic pathways as broad-spectrum antiviral strategies. Understanding this interconnected network will advance our knowledge of viral pathogenesis and identify new avenues for host-directed antiviral intervention.

## 1. Introduction

Flaviviruses are a group of positive-sense single-stranded RNA (+ssRNA) viruses belonging to the *Flaviviridae* family, which includes important human pathogens such as dengue virus (DENV), Zika virus (ZIKV), West Nile virus (WNV), yellow fever virus (YFV), hepatitis C virus (HCV), and Japanese encephalitis virus (JEV). These viruses share similar genomic organization and replication strategies but differ markedly in tissue tropism and disease outcomes, ranging from asymptomatic infections to severe hemorrhagic fever, encephalitis, and congenital or cardiac pathologies [1,2,3,4]. Despite these differences, a unifying hallmark of flavivirus replication is their reliance on host endoplasmic reticulum (ER) membranes for the formation of viral replication compartments (RCs), virion assembly, and protein processing [2,5,6].

**Figure 1:**
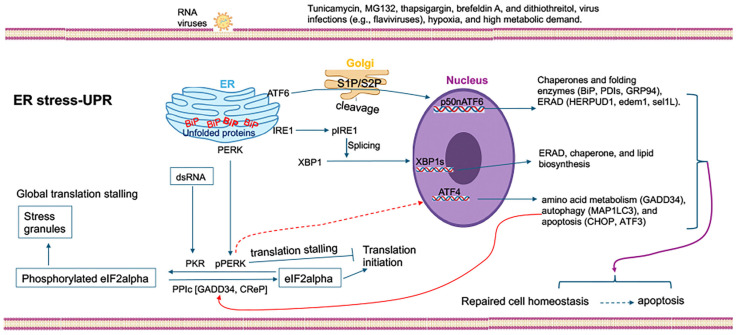
**ER remodeling and UPR activation during flavivirus infection and cellular stress.** Flavivirus infection, along with other cellular stressors, drives extensive remodeling of the endoplasmic reticulum (ER) and activates the unfolded protein response (UPR). Viral replication occurs on ER-derived membranes, where synthesis of the viral polyprotein and accumulation of viral proteins increase membrane demand and impose protein-folding stress. These conditions initiate UPR signaling through three canonical branches: PERK, IRE1, and ATF6. PERK activation leads to phosphorylation of eIF2α and induction of ATF4-dependent stress responses. IRE1 promotes splicing of XBP1 mRNA, resulting in transcriptional programs that support ER expansion, protein folding, and lipid metabolism. ATF6 is transported to the Golgi, where proteolytic cleavage releases its active form to drive expression of ER chaperones. Collectively, these pathways reprogram ER function to increase folding capacity, lipid synthesis, and membrane biogenesis, creating conditions that support viral replication while preserving ER homeostasis.

The ER is a central organelle that coordinates protein synthesis, folding, and trafficking while maintaining lipid and calcium homeostasis. Perturbations of ER function caused by excessive protein synthesis, lipid imbalance, or viral infection lead to the accumulation of misfolded proteins, a condition known as ER stress [7,8,9]. To restore homeostasis, cells activate a conserved signaling network called the unfolded protein response (UPR) [10]. The UPR is mediated by three ER transmembrane sensor pathways: protein kinase RNA-like ER kinase (PERK), inositol-requiring enzyme 1 (IRE1), and activating transcription factor 6 (ATF6) which initiate adaptive transcriptional and translational programs to increase protein folding capacity, enhance ER-associated degradation (ERAD), and restore metabolic balance [7,10]. However, if ER stress persists, the UPR shifts toward pro-apoptotic signaling to eliminate irreversibly damaged cells (Figure 1).

RNA viruses that replicate at the ER, such as flaviviruses, and coronaviruses, profoundly remodel ER membranes and subvert UPR signaling to support their life cycles [6,11,12]. During flavivirus infection, the synthesis of viral structural and nonstructural proteins imposes a heavy folding load on the ER, while replication complex formation and virion budding distort the ER architecture. Consequently, all three UPR branches are activated to varying degrees [13,14,15,16,17]. For example, DENV and ZIKV infections induce phosphorylation of eIF2α through PERK activation [15,18], leading to transient translation attenuation and selective expression of ATF4-dependent genes. The IRE1–XBP1 axis is activated to enhance ERAD and lipid biosynthesis, whereas ATF6 cleavage promotes chaperone expression to assist viral protein folding. Intriguingly, flaviviruses can fine-tune the timing and intensity of UPR activation enhancing adaptive responses that favor replication while suppressing terminal apoptotic outcomes that would limit viral propagation [15].

Beyond its canonical role in proteostasis, the UPR is now recognized as a key regulator of cellular metabolism, particularly lipid biosynthesis and lipid droplet (LD) formation [19,20]. LDs are dynamic lipid storage organelles that originate from the ER and are composed of a neutral lipid core surrounded by a phospholipid monolayer and associated proteins [21]. They serve as metabolic hubs that integrate lipid storage, signaling, and energy supply. During infection, LDs can act as scaffolds for viral protein assembly, platforms for immune signaling, or sources of lipids for membrane biogenesis [21]. Flaviviruses exploit LDs in multiple ways: DENV NS4A and NS4B interact with LD-associated proteins to promote replication complex formation [22], while ZIKV modulates LD metabolism differently depending on host cell type [21] either inducing accumulation or depletion to support replication and evade immune responses.

The interplay between ER stress, UPR, and LD metabolism represents a critical determinant of flavivirus infection outcomes [23]. The UPR regulates the transcription of key lipogenic enzymes including acetyl-CoA carboxylase (ACC), fatty acid synthase (FASN), and diacylglycerol acyltransferase (DGAT), thereby promoting LD biogenesis [24,25]. Conversely, perturbation of LD metabolism feeds back to exacerbate ER stress and alter UPR signaling. Infected cells thus enter a complex metabolic reprogramming state that supports viral replication but compromises organelle function, leading to inflammation, mitochondrial dysfunction, and apoptosis. In this context, the ER–LD interface functions not only as a replication hub but also as a metabolic checkpoint that determines cell survival or death [26,27].

In this review, we integrate current knowledge of how flavivirus infection triggers and exploits ER stress and the UPR, with an emphasis on their interplay with LD dynamics. We first provide a mechanistic overview of the UPR pathways and their regulation. We then summarize evidence of UPR activation and lipid remodeling during flavivirus infection, highlighting how these processes converge to facilitate replication and pathogenesis. Finally, we discuss therapeutic strategies targeting UPR–lipid metabolism interactions as a potential avenue for broad-spectrum antiviral intervention. Our recent findings reveal that ZIKV infection induces ER remodeling, mitochondrial fragmentation, and LD depletion in all permissive cells, suggesting a ZIKV-induced ER–LD interaction.

## 2. The Unfolded Protein Response (UPR): Mechanisms and Regulation

The ER serves as the principal site of protein folding and lipid synthesis in eukaryotic cells. Its ability to maintain homeostasis is essential for the secretory pathway and for the biosynthesis of cellular membranes. Perturbations that disrupt protein folding—such as viral protein overproduction, calcium imbalance, oxidative stress, or lipid perturbation lead to the accumulation of unfolded or misfolded proteins within the ER lumen, collectively referred to as ER stress [28,29]. To counteract this, cells activate an adaptive signaling network known as the UPR, which aims to restore ER function by temporarily halting protein translation, increasing the folding capacity, and enhancing degradation of misfolded proteins through the ER-associated degradation (ERAD) pathway [30,31,32,33].

A wide array of physiological, pathological, and environmental stimuli can activate the UPR [8]. Beyond viral infection, hypoxia, and high metabolic demand, UPR activation can be triggered by nutrient deprivation, oxidative stress, lipid overload, and inflammatory cytokines. Numerous pharmacological agents are also known to disturb ER function [34]. Thapsigargin depletes ER calcium stores by inhibiting SERCA pumps, brefeldin A blocks ER–Golgi trafficking, and dithiothreitol disrupts disulfide bond formation. Additional ER stressors including tunicamycin inhibiting N-linked glycosylation, proteasome inhibitors such as MG132 or bortezomib, and environmental toxins further illustrate the diverse mechanisms by which ER proteostasis can be perturbed. Collectively, these factors underscore the sensitivity of the UPR to disruptions in protein folding, redox balance, lipid homeostasis, and cellular signaling. The UPR is initiated by three ER-resident transmembrane sensors: protein kinase RNA-like endoplasmic reticulum kinase (PERK), inositol-requiring enzyme 1 (IRE1), and activating transcription factor 6 (ATF6), each responding to the accumulation of unfolded proteins through distinct but interconnected signaling cascades [8] (Figure 1). Under non-stressed conditions, these sensors are maintained in an inactive state through their interaction with the ER chaperone Binding immunoglobulin protein (BiP), also known as GRP78. During ER stress, BiP preferentially binds to misfolded proteins, leading to the dissociation and activation of PERK, IRE1, and ATF6. The subsequent activation of these pathways orchestrates a multifaceted transcriptional and translational program that determines whether the cell adapts to stress or undergoes apoptosis [8].

### 2.1. The PERK–eIF2α–ATF4 Axis: Translational Control and Metabolic Adaptation

**Figure 2:**
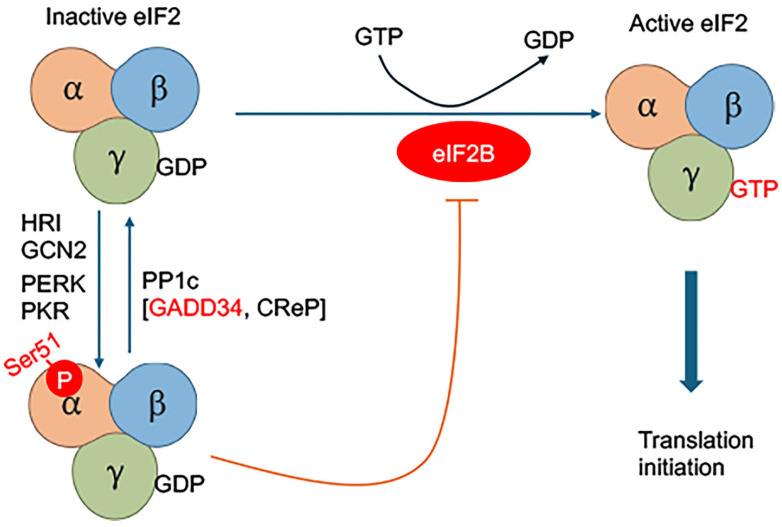
**Control of protein translation through eIF2α phosphorylation during ER stress.** The translation initiation factor eIF2 is required for delivery of initiator Met-tRNAi to the ribosome in its active GTP-bound form. Under basal conditions, eIF2–GTP assembles into a ternary complex with Met-tRNAi to initiate translation. Following start codon recognition, GTP is hydrolyzed to GDP, and eIF2 must be recycled to its active form by the guanine nucleotide exchange factor eIF2B. During ER stress, activation of kinases such as PERK leads to phosphorylation of eIF2α. This modification inhibits eIF2B activity, limiting GDP–GTP exchange, and reducing formation of the eIF2–GTP ternary complex. Consequently, global cap-dependent translation is suppressed, decreasing the influx of newly synthesized proteins into the ER and alleviating protein-folding stress.

**Figure 3:**
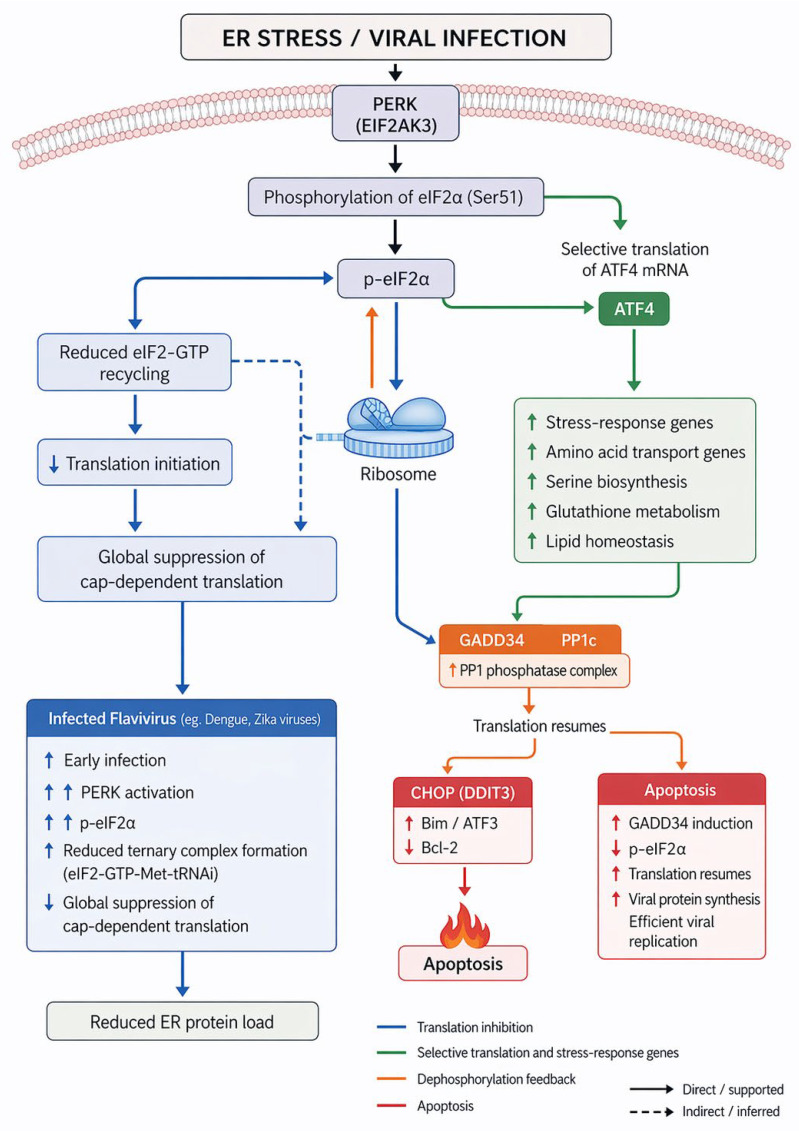
**PERK–eIF2α–ATF4 signaling regulates translational control and cellular adaptation during ER stress.** In response to ER stress or viral infection, the ER-resident kinase PERK (EIF2AK3) phosphorylates eIF2α at Ser51. Phosphorylated eIF2α inhibits eIF2B, impairing recycling of eIF2–GDP to eIF2–GTP and reducing assembly of the translation initiation complex. This results in broad suppression of cap-dependent protein synthesis, limiting ER protein load. Despite this reduction, select transcripts containing upstream open reading frames, including ATF4, are preferentially translated. ATF4 drives expression of genes involved in amino acid metabolism, redox balance, and stress adaptation. Under sustained stress, downstream effectors such as CHOP (DDIT3) promote apoptosis, whereas GADD34–PP1 complexes facilitate eIF2α dephosphorylation to restore translation. Several flaviviruses, including Dengue and Zika viruses, transiently engage in this pathway to modulate host translation and support viral replication.

PERK (EIF2AK3) is a type I transmembrane kinase that phosphorylates the α-subunit of eukaryotic initiation factor 2 (eIF2α) upon activation. Other proteins phosphorylating the eIF2α include Heme-Regulated Inhibitor (HRI), General Control Nonderepressible 2 (GCN2), and Protein Kinase R (PKR) (Figure 2). Phosphorylated eIF2α (p-eIF2α) inhibits the guanine nucleotide exchange factor eIF2B, resulting in global attenuation of cap-dependent protein translation [35] (Figure 2). This translational repression reduces the burden on the ER by limiting the influx of nascent polypeptides. However, specific mRNAs containing upstream open reading frames (uORFs), such as ATF4, are preferentially translated under these conditions [35,36] (Figure 2 and Figure 3).

ATF4 acts as a master transcription factor that induces genes involved in amino acid transport, antioxidant defense, autophagy, and stress recovery [37]. Among its targets are CHOP (C/EBP homologous protein, also known as GADD153 or DDIT3) and GADD34, which form feedback loops that fine-tune UPR signaling. CHOP promotes apoptosis by inducing pro-apoptotic genes such as Bim and ATF3, and by suppressing anti-apoptotic proteins such as Bcl-2. In contrast, GADD34, in complex with protein phosphatase 1 (PP1c), dephosphorylates eIF2α to resume protein synthesis once ER homeostasis is restored [38,39] (Figure 2 and Figure 3).

The PERK pathway also intersects with cellular metabolism. ATF4 transcriptionally regulates enzymes involved in serine biosynthesis, glutathione metabolism, and lipid homeostasis, thereby linking the UPR to redox balance and metabolic adaptation [40]. In the context of viral infection, PERK activation can restrict viral replication by inhibiting translation, but many viruses including flaviviruses, exploit transient PERK activation to modulate host translation while ensuring selective synthesis of viral proteins [40].

For instance, DENV and ZIKV infections activate PERK early during infection, resulting in phosphorylation of eIF2α and transient suppression of host translation [15,40]. This promotes stress granule formation, which sequesters antiviral mRNAs and delays innate immune activation. Subsequently, the viruses utilize GADD34-dependent dephosphorylation of eIF2α to resume translation and facilitate viral protein synthesis [41]. Thus, the PERK–eIF2α–ATF4 axis operates as a double-edged sword: it initially limits viral protein load but is later co-opted to support viral replication and survival.

### 2.2. The IRE1–XBP1 Pathway: Signaling for ER Expansion and Lipid Synthesis

**Figure 4:**
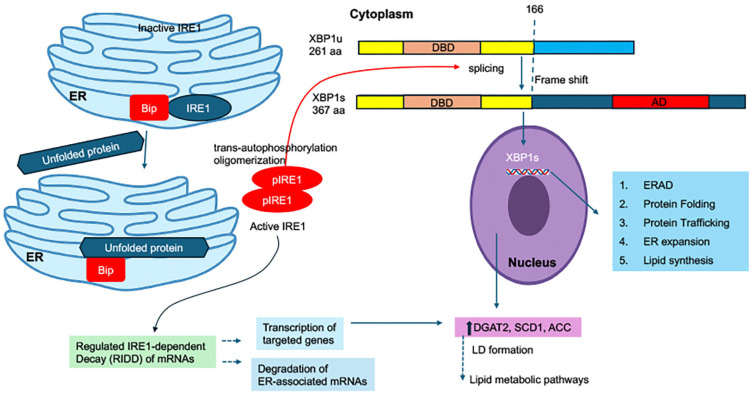
**The IRE1–XBP1 branch of the unfolded protein response.** Accumulation of unfolded proteins within the ER lumen activates the stress sensor IRE1. Under basal conditions, IRE1 is maintained in an inactive state through association with the chaperone BiP/GRP78. During ER stress, BiP dissociates from IRE1 and preferentially binds misfolded proteins, permitting IRE1 oligomerization and trans-autophosphorylation, which in turn activates its endoribonuclease domain. Activated IRE1 mediates the unconventional splicing of XBP1 mRNA by excising a 26-nucleotide intron, producing the spliced isoform XBP1s. The translated XBP1s protein acts as a transcription factor that translocates to the nucleus and induces expression of genes involved in protein folding, ER-associated degradation (ERAD), lipid biosynthesis, and ER membrane expansion, thereby promoting restoration of ER homeostasis. In parallel with XBP1 mRNA splicing, activated IRE1 can initiate regulated IRE1-dependent decay (RIDD), selectively degrading ER-localized mRNAs to limit the influx of nascent proteins into the ER. Together, these coordinated outputs of IRE1 signaling facilitate cellular adaptation to ER stress.

IRE1 (inositol-requiring enzyme 1) is the most evolutionarily conserved UPR sensor and possesses both serine/threonine kinase and endoribonuclease (RNase) activities. Upon activation, IRE1 oligomerizes and undergoes trans-autophosphorylation, which triggers its RNase domain to excise a 26-nucleotide intron from X-box binding protein 1 (XBP1) mRNA. The spliced form, XBP1s, encodes a potent transcription factor that translocates to the nucleus and activates genes involved in ER biogenesis, ERAD, protein folding, and lipid biosynthesis [42] (Figure 4).

Through XBP1s, the IRE1 pathway orchestrates ER membrane expansion and promotes lipid metabolic pathways necessary for organelle remodeling. XBP1s upregulate key lipogenic genes such as DGAT2, SCD1, and ACC, directly linking UPR signaling to LD formation and phospholipid synthesis [43,44]. This pathway thereby provides the structural and metabolic foundation for sustaining ER function during increased protein synthesis (Figure 4).

Beyond XBP1 splicing, activated IRE1 can degrade selected mRNAs and microRNAs through a process known as regulated IRE1-dependent decay (RIDD) [45] (Figure 4). RIDD reduces the load of secretory proteins entering the ER but can also modulate antiviral responses by degrading transcripts related to immune signaling. For example, RIDD-mediated degradation of antiviral mRNAs, such as those encoding interferon-stimulated genes (ISGs), has been observed in certain viral infections, allowing viruses to evade innate immunity.

### 2.3. The ATF6 Pathway: Induction of Chaperones and Folding Enzymes

**Figure 5:**
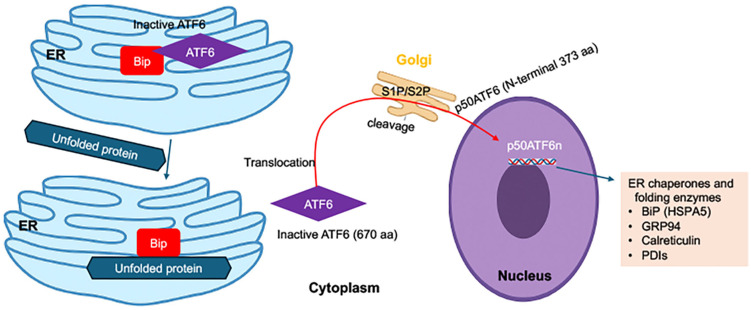
**The ATF6 branch of the unfolded protein response (UPR).** Under basal conditions, the ER stress sensor ATF6 is embedded in the ER membrane in an inactive state through its association with the chaperone BiP/GRP78. During ER stress, accumulation of unfolded proteins in the ER lumen sequesters BiP away from ATF6, enabling ATF6 to dissociate and traffic to the Golgi apparatus. In the Golgi, ATF6 undergoes sequential proteolytic processing by site-1 and site-2 proteases (S1P and S2P), releasing the cytosolic, transcriptionally active fragment p50ATF6. This fragment translocates to the nucleus, where it induces expression of genes encoding ER chaperones and protein-folding machinery, including BiP (HSPA5), GRP94, calreticulin, and protein disulfide isomerases (PDIs). ATF6 also cooperates with XBP1s to upregulate components of the ER-associated degradation (ERAD) pathway, thereby enhancing protein-folding capacity and preserving ER proteostasis under conditions of cellular stress.

ATF6 is a type II transmembrane protein that, under normal conditions, resides in the ER membrane as an inactive precursor. Upon ER stress, ATF6 (670 aa) dissociates from BiP and translocates to the Golgi apparatus, where it is sequentially cleaved by site-1 protease (S1P) and site-2 protease (S2P). This cleavage releases the cytosolic fragment p50ATF6 (N terminal 373 aa of ATF6), a basic leucine zipper (bZIP) transcription factor that enters the nucleus and activates genes encoding ER chaperones and folding enzymes such as BiP, GRP94, calreticulin, and protein disulfide isomerases (PDIs) [46,47,48] (Figure 5).

The ATF6 branch thus primarily enhances the protein folding capacity and quality control machinery of the ER, complementing the functions of PERK and IRE1 pathways. In addition, ATF6 synergizes with XBP1s to induce genes involved in ERAD and lipid metabolism, contributing to ER homeostasis and structural reorganization [49].

Flaviviruses appear to utilize ATF6 activation to support their replication cycles. For example, DENV infection increases ATF6 cleavage and upregulates chaperones such as BiP and GRP94, which assist in the folding and maturation of viral proteins [50]. ZIKV infection similarly activates ATF6 early after infection, correlating with elevated expression of ER chaperones and remodeling of ER membranes [17,51]. Interestingly, some viral proteins, including DENV NS4B and HCV E2, can directly modulate ATF6 signaling to enhance viral protein folding while preventing the activation of apoptotic downstream responses [52,53].

### 2.4. Crosstalk and Integration Among UPR Branches

Although PERK, IRE1, and ATF6 initiate distinct signaling cascades, they function in an integrated and temporally regulated manner. During early ER stress, all three sensors cooperate to promote cell survival: PERK attenuates translation, IRE1 enhances folding and lipid synthesis, and ATF6 increases chaperone production. If stress persists, feedback loops involving CHOP, GADD34, and JNK convert the adaptive response into apoptosis [54].

Importantly, there is extensive cross-regulation among these branches. ATF4 and XBP1s share overlapping target genes involved in lipid metabolism and autophagy. XBP1s can stabilize ATF6 signaling by promoting ER expansion, while prolonged PERK activation suppresses IRE1 signaling to prevent overactivation. The outcome of UPR activation thus depends on the intensity and duration of stress, as well as the cellular context [55,56].

In viral infections, this crosstalk is finely tuned by viral proteins to maximize replication efficiency while minimizing cytotoxicity. For example, flaviviruses may sequentially activate UPR branches, initially triggering PERK and ATF6 for adaptive responses, followed by selective suppression of IRE1-mediated apoptosis to maintain a favorable environment for replication. This dynamic interplay underscores the versatility of the UPR as both a cellular defense mechanism and a viral exploitation target [56,57].

## 3. Flavivirus-Induced ER Remodeling and UPR Activation

Flaviviruses are obligate intracellular pathogens that rely heavily on the host ER for replication, polyprotein processing, and virion assembly [21,58,59]. Upon infection, these viruses extensively remodel the ER into specialized membranous structures that serve as replication compartments (RCs), shielding viral RNA intermediates from cytosolic sensors and providing an optimal lipid environment for RNA synthesis. However, this massive rearrangement of the ER membrane and the overwhelming production of viral proteins impose significant folding stress, inevitably activating the UPR.

**Figure 6:**
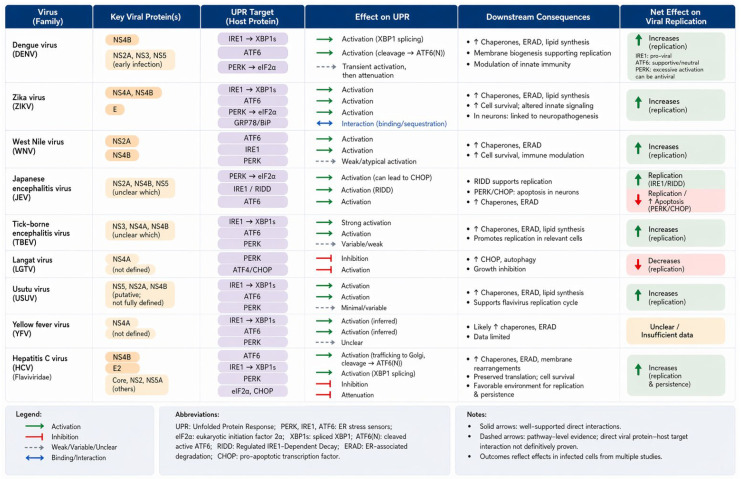
**Interactions of orthoflaviviruses, Usutu virus, and hepatitis C virus with UPR pathways and their impact on viral replication.** This figure summarizes the interactions between representative orthoflaviviruses—including dengue virus (DENV), Zika virus (ZIKV), West Nile virus (WNV), Japanese encephalitis virus (JEV), tick-borne encephalitis virus (TBEV), Langat virus (LGTV), Usutu virus (USUV), and yellow fever virus (YFV)—as well as hepatitis C virus (HCV), and the three major branches of the unfolded protein response (UPR): PERK–eIF2α, IRE1–XBP1, and ATF6 pathways. For each virus, the table lists key viral proteins implicated in UPR modulation, their reported host UPR targets, and the resulting effects on UPR signaling, including activation, inhibition, or modulation of specific pathways. Downstream consequences of UPR engagement are summarized, including induction of chaperones and ER-associated degradation (ERAD), lipid biosynthesis and membrane remodeling, regulation of autophagy and apoptosis, and modulation of innate immune responses. The net effects on viral replication are indicated, highlighting whether UPR activation promotes or restricts infection. In most flaviviruses, activation of the IRE1–XBP1 and ATF6 pathways supports viral replication by enhancing ER folding capacity and lipid metabolism required for replication compartment formation. In contrast, sustained activation of the PERK–eIF2α–ATF4/CHOP axis may exert antiviral effects by inducing translational arrest or apoptosis, as observed in certain contexts such as JEV or LGTV infection. HCV is included as a well-characterized model illustrating direct viral protein interactions with UPR components, including NS4B-mediated activation of ATF6 and IRE1 pathways and E2-mediated modulation of PERK signaling. Evidence levels are indicated to distinguish between direct protein–protein interactions and pathway-level observations inferred from infection studies. Arrows denote the direction and nature of regulation: activation (green), inhibition (red), and indirect or context-dependent effects (dashed lines). Overall, this figure highlights both conserved and virus-specific strategies by which flaviviruses exploit UPR signaling to optimize replication while balancing host cell stress responses.

UPR activation during flavivirus infection is not merely a collateral consequence of ER stress, but a finely tuned process orchestrated by the virus to balance ER homeostasis, lipid biosynthesis, and immune evasion. Evidence from multiple flaviviruses including DENV, ZIKV, TBEV, HCV, WNV, JEV, YFV, and Usutu virus (USUV) demonstrates that each branch of the UPR (PERK, IRE1, and ATF6) is differentially activated and temporally regulated to support distinct stages of the viral life cycle (Figure 6).

### 3.1. ER Remodeling as a Hallmark of Flavivirus Replication

Following entry and uncoating, the flavivirus positive-sense RNA genome is translated into a single polyprotein that is co- and post-translationally processed by host and viral proteases into three structural (C, prM/M, E) and seven nonstructural (NS1–NS5) proteins [60]. Most nonstructural proteins are membrane-associated and localize to the ER, where they orchestrate the formation of replication organelles clusters of invaginated vesicles and convoluted membranes often referred to as vesicle packets or virus-induced RCs [61,62].

These RCs are characterized by extensive ER rearrangements and are physically continuous with the ER lumen, indicating that they arise from ER-derived membranes. Ultrastructural analyses of DENV- and ZIKV-infected cells reveal that the RCs are composed of double-membrane vesicles (~80–100 nm in diameter) connected to the cytoplasm by neck-like channels that allow the exchange of metabolites and newly synthesized viral RNA [62,63]. This architectural remodeling is driven largely by viral nonstructural proteins, particularly NS4A, NS4B, and NS1, which modulate ER curvature and membrane proliferation.

Such remodeling places immense pressure on ER homeostasis. The increased demand for membrane lipids, combined with the accumulation of misfolded viral proteins, triggers ER stress and subsequent UPR activation. Interestingly, the nature and magnitude of UPR activation vary among flaviviruses and depend on cell type, infection stage, and viral load. Early activation of adaptive UPR signaling supports viral replication by enhancing chaperone availability and lipid synthesis, whereas prolonged or excessive activation can lead to apoptosis and limit viral spread.

### 3.2. Zika Virus (ZIKV): Integrated UPR Activation and Organelle Remodeling

ZIKV infection activates all three UPR branches (PERK, IRE1, and ATF6) [17,18], although activation of the ATF6 pathway remains somewhat debated [64]. ZIKV activates PERK signaling in diverse cell types, including neural progenitor, placental, A549, Huh7, and cardiac cells [17,18,65,66,67,68]. This pathway activation leads to PERK–eIF2α signaling, which induces transient translational arrest and activation of ATF4/CHOP, while viral proteins NS4A and NS4B modulate PERK and mTOR signaling to maintain selective viral translation [69]. ZIKV-induced eIF2α phosphorylation correlates with increased ATF4 and CHOP expression, suggesting activation of the integrated stress response (ISR). In neural progenitor cells, this delicate balance contributes to cell cycle arrest and differentiation defects associated with ZIKV-induced microcephaly [17].

ZIKV also engages the IRE1–XBP1 pathway, though with distinct kinetics and intensity [17,70], promoting ER expansion [68], lipid remodeling [68], and in some contexts, RIDD-mediated suppression of antiviral transcripts [71]. In ZIKV-infected neural and cardiac cells, XBP1 splicing correlates with upregulation of ER chaperones (BiP, GRP94) and lipid metabolic genes, contributing to both ER expansion and lipid droplet (LD) remodeling [68]. Interestingly, prolonged IRE1 activation in ZIKV infection may also mediate RIDD (regulated IRE1-dependent decay) activity, which degrades select host mRNAs involved in innate immune signaling, thereby potentially dampening antiviral responses.

ATF6 activation further enhances chaperone production and ER folding capacity. Notably, ZIKV induces pronounced mitochondrial fragmentation and lipid droplet depletion in certain cell types, reflecting a unique UPR–lipid–organelle remodeling phenotype linked to neuropathogenesis and cardiotoxicity. Likewise, ZIKV infection activates ATF6 early post-infection, correlating with enhanced chaperone expression and ER membrane proliferation [17].

Another cellular response to external stressors is the formation of stress granules (SG). However, ZIKV infection not only failed to induce SG formation but also suppresses SG formation induced by stressors such as sodium arsenite, poly(I·C) and hippuristanol [66]. Further experiments demonstrated that ZIKV capsid protein, NS3/NS2B-3, and NS4A of ZIKV interfered with SG formation [66]. Another study reported that NS2B interacts with protein phosphatase 1α (PP1α) to inhibit SG formation [72]. This inhibition of SG formation has also been reported in other flaviviruses, including Usutu virus [73].

### 3.3. Dengue Virus (DENV): Dynamic Regulation of UPR to Optimize Replication

It was reported in 2006 that both DENV-2 and Japanese encephalitis virus (JEV) infections activate the XBP1 pathway, as evidenced by XBP1 splicing and XBP1s protein expression [74]. NS2B of DENV-2, but not of JEV, is a potential protein inducer of XBP1 splicing. In DENV-infected hepatocytes, PERK autophosphorylation and eIF2α phosphorylation occur within hours of viral entry, coinciding with increased expression of ATF4 and its downstream targets CHOP and GADD34 [15,75]. This transient translational arrest reduces global protein synthesis, thereby alleviating ER overload, while selectively enhancing the translation of stress-responsive transcripts that promote cell survival. DENV-2 activation of UPR is time-dependent. In the first 6 h post infection of human fibrosarcoma cell line 2fTGH with DENV-2, early activation of PERK leads to eIF2α phosphorylation and transient translational attenuation, followed by strong activation of XBP1 pathway, leading to GADD34-mediated recovery of protein synthesis [15]. It is hypothesized that time-dependent activation of the UPR by DENV-2 can override translational inhibition, prevent apoptosis, and favor viral replication [75].

The effects of UPR on viral replication occur at two levels. Functional studies using PERK inhibitors or eIF2α phosphatase inhibitors demonstrate that moderate PERK activation supports viral replication, whereas sustained activation that maintains eIF2α phosphorylation suppresses viral protein synthesis and limits infection [76,77,78]. During DENV infection, ATF6 cleavage and nuclear translocation occur concurrently with BiP and GRP94 upregulation, facilitating the folding of viral E and prM proteins [15,79,80]. Inhibition of ATF6 activation impairs DENV protein maturation and virion assembly, highlighting its supportive role in infection. This effect was also demonstrated using VER-155008, a Bip inhibitor [81]. However, UPR activation has also been reported to contribute to innate defense against viral replication. For example, ER stress in HepG2 cells caused by DENV-2 is coupled with the induction of multiple cell death pathways as a mechanism to eliminate infected liver cells [82]. It has also been reported that flaviviruses, including Dengue, Zika, West Nile and Tick-borne encephalitis viruses, activate the UPR, promoting transcription of interferon regulatory factor 3 induced antiviral genes [83].

Flaviviruses have evolved multiple strategies to manipulate UPR signaling. Several nonstructural proteins interact directly with UPR sensors or their regulators. DENV NS4A and NS4B induce ER membrane curvature and activate PERK and ATF6 signaling while inhibiting excessive IRE1 activation [15,84]. NS2B3 protease can cleave BiP or other host factors to modulate ER stress signaling [85]. ZIKV NS4A and NS4B suppress the Akt–mTOR pathway, enhancing autophagy and maintaining metabolic balance during ER stress [69]. Moreover, NS1, a secreted glycoprotein, contributes to ER remodeling and lipid trafficking, indirectly influencing UPR activation [86]. For instance, DENV NS4B can suppress excessive ATF6 activation to prevent apoptosis while maintaining sufficient chaperone induction for viral protein processing [52,84,87]. Therefore, targeting flavivirus-UPR interaction may provide opportunities to develop broad-spectrum antiviral strategies [88].

UPR activation during DENV infection is also involved in viral pathogenesis. It has been observed that UPR gene expression in THP-1 cells during antibody-dependent enhancement (ADE) infection correlates with DENV-2-caused disease severity [89]. Celgosivir, a small molecular drug, induces DENV NS1 protein misfolding to cause UPR activation and protects against lethal challenge with DENV-2 in a mouse model [90]. Another comprehensive in vivo and in vitro study revealed that DENV-2-induced ER stress increases autophagy activity, viral replication, and pathogenesis through two UPR signaling pathways: PERK-eIF2α and IRE1α-JNK [77]. Unfortunately, most studies regarding the UPR are based on DENV-2 infection models, although one study reported differential UPR between DENV-1 and DENV-2 [91]. The roles of DENV proteins are also not completely understood, which is an important area for developing targeted antiviral strategies.

### 3.4. West Nile Virus (WNV): Engagement of UPR Pathways

WNV activates all three UPR signaling pathways. Unlike in DENV-2 infected cells, in WNV-infected neuronal cells the XBP1 pathway appears to be dispensable, while PERK signaling induces transient but prolonged eIF2α phosphorylation, leading to CHOP production. CHOP is responsible for premature cell death and host defense against viral replication. Therefore, WNV infection-caused UPR is also responsible for widespread neuronal loss in infected neuronal tissue [13]. Another study showed that WNV preferentially utilizes the IRE1–XBP1 and ATF6 pathways to support ER remodeling and replication, while PERK-eIF2a activation is relatively limited [92]. ATF6 signaling is required to promote cell survival and inhibit innate immune responses, allowing WNV to replicate efficiently [93]. This selective engagement of UPR branches may help WNV evade host immune responses while maintaining sufficient ER function for replication.

Interestingly, WNV and JEV infections also activate PERK signaling, but their downstream effects differ. In WNV-infected cells, eIF2α phosphorylation transiently suppresses host translation but does not inhibit viral protein synthesis, suggesting that viral RNAs may bypass host translational control [13]. In JEV-infected neurons, PERK–CHOP signaling contributes to apoptosis and neurodegeneration at late stages, indicating a context-dependent transition from adaptive to pro-apoptotic outcomes [94].

To determine whether specific WNV proteins are associated with UPR induction, non-structural proteins NS4A and NS4B have been identified as independently activating UPR and inducing autophagy [92,95]. However, it is not fully understood how these proteins interact with UPR-associated factors or whether UPR activation facilitates proper folding of WNV protein in the ER.

### 3.5. Tick-Borne Encephalitis Virus (TBEV): IRE1-Driven Lipid and ER Remodeling

TBEV, the causative agent of Tick-borne encephalitis (TBE) which is a serious human neurological disease, strongly activates the IRE1–XBP1 pathway, which promotes ER membrane expansion and lipid biosynthesis required for replication. TBEV also activates the ATF6 pathway, as evidenced by ATF6 translocation to the nucleus and cleavage of ATF6 [96]. PERK signaling is also engaged but may play a more regulatory or restrictive role depending on the cellular context [83]. The dependence of TBEV on IRE1 signaling highlights the importance of lipid metabolic reprogramming in supporting viral replication, particularly in neuronal and endothelial cells. Furthermore, other tick-borne flaviviruses (TBFVs), including Langat virus, strongly activate IRE1-XBP1 pathway in a cell-type-dependent manner to promote viral replication [97]. Interestingly, Langat virus-induced PERK-mediated UPR signaling restricts viral replication [98].

### 3.6. Japanese Encephalitis Virus (JEV): UPR Activation and Neurotoxicity

JEV infection induces robust ER stress and activates all three UPR branches. PERK–CHOP signaling contributes to apoptosis and neurodegeneration in infected neurons, linking UPR activation to disease pathology [99,100,101]. JEV triggers the IRE1 pathway, including RIDD activity, which may support viral replication by modulating host mRNA stability and immune signaling while helping to alleviate virus-induced cytotoxicity [74,102,103,104]. ATF6 contributes to chaperone induction and ER homeostasis, although it is less well studied in JEV infection models. More recently, it was demonstrated that Poly ADP-ribose polymerase-16 (PARP-16) regulates JEV-mediated ER stress response (PERK and IRE1 pathways) in neural stem/progenitor cells (NSPCs) in the BALB/c mouse model. The siRNA-mediated in vitro downregulation of PARP-16 in NSPCs alleviated overall UPR activation, highlighting its role during JEV infection. Thus, JEV exemplifies the dual role of UPR in promoting replication while contributing to tissue damage.

### 3.7. Usutu Virus (USUV): Emerging Evidence of UPR Activation

USUV, an emerging neurotropic flavivirus, has been shown to induce ER stress and activate UPR signaling, although mechanistic studies remain limited. Available evidence suggests engagement of the IRE1 pathway, with potential roles in ER remodeling and viral replication [105]. Similar to ZIKV [66], USUV infection suppresses the SG formation induced by stressors such as the HRI activator ArsNa [73]. Given its close phylogenetic relationship to WNV, USUV may share similar strategies in selectively modulating UPR branches. Further investigation is needed to define the specific viral proteins involved and their impact on host lipid metabolism and immune responses.

### 3.8. Hepatitis C Virus (HCV): Prototypic Manipulation of UPR Signaling

HCV, although classified separately within the Hepacivirus genus, provides one of the most mechanistically defined examples of UPR manipulation among flavivirus-related viruses. Early study using an HCV replicon composed of the HCV 5-NCR, encephalomyocarditis virus IRES, and coding region of NS3 to NS5B to infect Huh7 cells demonstrated activation of ATF6 transactivation activity and increased GRP78 (BiP) expression [106]. HCV subgenomic replicons may suppress the IRE1-XBP1 pathway to enhance viral expression in hepatocytes [107]. HCV envelope proteins E1 and E2 regulate CHOP expression in a PERK-dependent manner, suggesting activation of PERK-ATF4 pathway [108,109]. HCV NS4B induces ATF6 activation and IRE1-XBP1 pathway, favoring viral replication [110]. These effects, including induction of XBP1 splicing and ATF6 cleavage, may also contribute to reactive oxygen species (ROS) production and inflammation [14]. However, the involvement of other HCV proteins in UPR activation remains unclear.

Autophagy is a cellular mechanism to maintain homeostasis and serves as innate immunity to remove invaded pathogens out of cells. HCV infection has been reported to induce incomplete autophagy in a UPR-dependent manner, as suppression of UPR pathways reduces LC3 lipidation [111]. Other studies report complete autophagy that promotes viral replication [112]. Additionally, HCV core protein activates autophagy through PERK–eIF2α and ATF6 pathways [113]. However, conflicting studies suggest that UPR is not required for HCV-induced autophagy, highlighting the need for further investigation [114]. More investigations are needed to clarify the discrepancies.

Using C57BL/6 transgenic mice expressing the HCV polyprotein, it was found that iron-induced UPR is responsible for fat accumulation in the liver, leading to hepatic steatosis in these transgenic mice [115]. The importance of UPR in HCV-caused liver pathogenesis is also supported by clinical evidence: livers from patients with untreated chronic hepatitis C exhibited in vivo hepatocyte ER stress and activation of the three UPR sensors [116]. However, the pathogenic role of UPR in HCV-related diseases has been challenged by another clinical study. This study demonstrated that downstream components of the UPR were not activated in patients with chronic HCV infection, suggesting that UPR may not play a major role in liver injury in these patients [117]. This observation is further supported by experimental results from a transgenic mouse model carrying the full-length HCV genome, which developed hepatic steatosis without detectable ER stress [118]. Therefore, the involvement of UPR in HCV pathogenesis requires more careful investigation.

### 3.9. Yellow Fever Virus (YFV): Limited but Conserved UPR Engagement

Although less extensively studied, YFV infection is thought to activate conserved UPR pathways similar to other flaviviruses. Evidence suggests engagement of the IRE1–XBP1 and ATF6 branches to support ER expansion and protein folding. However, the specific viral proteins and mechanisms that modulate UPR signaling in YFV remain poorly defined. Further studies are needed to determine whether YFV employs distinct strategies or shares common mechanisms with other flaviviruses.

### 3.10. Temporal and Spatial Coordination of UPR Activation, and Viral Modulation and Evasion of UPR Signaling

UPR activation during flavivirus infection is tightly regulated in both spatial and temporal dimensions. Early during infection, mild ER stress triggers adaptive UPR signaling that promotes lipid synthesis, chaperone production, and ER expansion that are conditions favorable for replication complex formation. As viral replication peaks, feedback mechanisms (e.g., GADD34-mediated eIF2α dephosphorylation or suppression of IRE1-mediated apoptosis) maintain ER homeostasis. In late infection, when viral protein accumulation exceeds the adaptive capacity, sustained CHOP and JNK activation lead to apoptosis, facilitating virus release and dissemination [119,120].

Spatially, UPR activation is localized around viral replication sites. Immunofluorescence and electron microscopy studies reveal colocalization of p-eIF2α, XBP1s, and BiP with DENV and ZIKV replication compartments [121,122,123], suggesting that UPR components are recruited to or activated near the ER-derived membranes containing viral proteins. This spatial coupling ensures localized adaptation of ER function to meet the metabolic and folding demands of viral replication.

These viral manipulations serve several purposes: (1) optimizing replication by enhancing chaperone-assisted folding and lipid supply; (2) preventing premature apoptosis that would curtail viral propagation; and (3) suppressing immune signaling by interfering with UPR–innate immunity cross-talk (e.g., IRE1–TRAF2–NF-κB axis). Through selective activation and suppression of UPR branches, flaviviruses achieve a fine balance between adaptation and cell death, ensuring efficient replication while minimizing host defense activation.

Collectively, evidence across flaviviruses demonstrates that the UPR is both a cellular defense mechanism and a viral exploitation target. The PERK, IRE1, and ATF6 branches are differentially activated to reshape the ER, augment lipid synthesis, and manage the folding load imposed by viral protein production. Temporal regulation allows the virus to harness early adaptive responses for replication and later limit pro-apoptotic signaling to sustain infection. Understanding how flaviviruses choreograph these UPR pathways provides a foundation for dissecting the downstream effects on LDs, organelle interactions, and metabolic reprogramming, which will be discussed in the next section.

## 4. Lipid Droplets (LDs) as Dynamic Organelles at the ER–UPR Interface

Lipid droplets (LDs) are increasingly recognized as central regulators at the intersection of endoplasmic reticulum (ER) homeostasis, unfolded protein response (UPR) signaling, and flavivirus replication. Because LD biogenesis originates from the ER membrane, and flaviviruses extensively remodel ER architecture to form replication compartments, a functional link between LD dynamics and UPR activation is mechanistically compelling. The IRE1–XBP1 axis of the UPR plays a pivotal role in this process by promoting lipid biosynthesis and ER membrane expansion, both of which are essential for the formation of viral replication compartments. Activation of XBP1s induces transcription of key lipogenic enzymes, including DGAT2, SCD1, and ACC, thereby increasing the synthesis of phospholipids and neutral lipids required for membrane proliferation. Consistent with this model, pharmacological inhibition or siRNA-mediated depletion of IRE1 or XBP1 significantly impairs Dengue virus (DENV) replication, underscoring the importance of UPR-driven lipid remodeling in establishing replication-competent membranes [80].

LDs are multifunctional organelles composed of a neutral lipid core, mainly triglycerides (TG) and cholesteryl esters (CE), surrounded by a phospholipid monolayer enriched in specific proteins. Beyond their canonical role in lipid storage, LDs serve as dynamic hubs integrating metabolism, stress signaling, and innate immunity [21,124,125]. Their ER origin and sustained physical association with ER membranes position LDs as critical platforms for viral exploitation during infection.

### 4.1. UPR Regulation of LD Biogenesis

**Figure 7:**
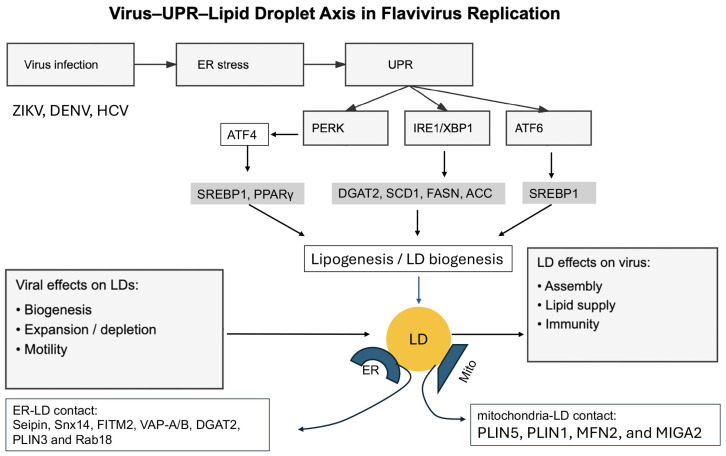
**UPR–lipid droplet crosstalk in flavivirus replication.** Flavivirus infection induces ER stress and activates the unfolded protein response (UPR), including PERK, IRE1, and ATF6 pathways. UPR signaling promotes lipogenesis and lipid droplet (LD) biogenesis through transcriptional programs involving XBP1s and ATF4, which induce key lipid metabolic enzymes such as DGAT1/2, SCD1, FASN, and ACC, as well as SREBP1 and PPARγ. LDs originate from the ER through neutral lipid accumulation and budding processes regulated by seipin and FIT proteins. LDs form membrane contact sites (MCSs) with the ER, mediated by VAP-A/B, Rab18, DGAT2, and SNARE proteins, enabling lipid exchange and replication compartment formation. Flaviviruses exploit LDs as platforms for virion assembly, lipid supply, and immune modulation, while also remodeling LD dynamics through lipolysis and redistribution. LDs reciprocally regulate ER stress by buffering lipids and proteotoxic stress. In ZIKV infection, excessive lipid consumption leads to LD depletion, mitochondrial dysfunction, and cell death, contributing to tissue-specific pathology.

LD biogenesis is initiated within the ER bilayer membrane, where neutral lipids synthesized by ER- resident enzymes such as diacylglycerol acyltransferase 1 and 2 (DGAT1/2) and acyl-CoA: cholesterol acyltransferase (ACAT) accumulate between the membrane leaflets. When the concentration of these neutral lipids exceeds a critical threshold, they nucleate to form lipid lenses that bud into the cytoplasm, encapsulated by a phospholipid monolayer continuous with the ER outer leaflet [21,124,125]. This process is spatially regulated by ER subdomains enriched in seipin, FITM1/2, and Pex30, which stabilize nascent LDs and coordinate their maturation. Once formed, LDs maintain physical and functional connectivity with the ER through membrane contact sites (MCSs) that allow bidirectional lipid and protein exchange. These ER–LD contacts are mediated by tethering proteins such as VAP-A/B, DGAT2, and Rab18, the latter linking LDs to the COPI machinery and autophagy-related processes [126,127,128,129,130]. Under homeostatic conditions, LD biogenesis buffers excess fatty acids and prevents lipotoxicity. However, under ER stress, the demand for neutral lipid storage increases, and the UPR transcriptionally reprograms lipid metabolism to promote LD formation both as a cytoprotective adaptation and as a structural foundation for viral replication (Figure 7).

Each branch of the UPR contributes to lipid metabolic reprogramming and LD biogenesis (Figure 7), albeit via distinct mechanisms. First, XBP1s is a master regulator of ER expansion and lipogenesis. It upregulates enzymes involved in phospholipid synthesis (e.g., choline cytidylyltransferase α, CCTα) and neutral lipid synthesis (DGAT2, SCD1, FASN) [43,131]. During flavivirus infection, XBP1s-driven lipid synthesis supplies membranes for replication organelles and precursors for LD biogenesis. DENV and ZIKV both induce XBP1s-dependent transcription of DGAT2 and FASN, correlating with increased LD abundance. Pharmacologic inhibition of IRE1 RNase activity or genetic silencing of XBP1 reduces LD accumulation and impairs viral replication, confirming the functional coupling between UPR-driven lipogenesis and virus propagation [74]. Second, ATF4 transcriptionally regulates genes involved in amino acid metabolism, antioxidant defense, and lipid biosynthesis. Under ER stress, ATF4 induces sterol regulatory element-binding protein 1 (SREBP1) and peroxisome proliferator-activated receptor gamma (PPARγ), two transcription factors critical for de novo lipogenesis and LD formation [132,133]. In DENV and ZIKV infection, PERK activation enhances SREBP1 cleavage and nuclear translocation, driving expression of fatty acid synthase (FASN), stearoyl-CoA desaturase (SCD1), and acetyl-CoA carboxylase (ACC) [65,134]. This cascade ensures a continuous supply of fatty acids for both membrane proliferation and neutral lipid synthesis. Thirdly, although primarily associated with ER chaperone induction, ATF6 also contributes to lipid homeostasis. Activated ATF6 can modulate SREBP1 processing and upregulate genes involved in phospholipid biosynthesis, thereby maintaining the structural integrity of the ER and LDs. Some evidence suggests that ATF6 interacts with CREB3L3 (Luman) to fine-tune lipid synthesis under stress conditions, a potential mechanism exploited by flaviviruses to stabilize ER–LD contacts [93,135]. Together, these pathways orchestrate a transcriptional and metabolic program that promotes LD biogenesis during flavivirus infection. Importantly, UPR-driven lipid synthesis not only expands the ER network but also enriches specific lipid species (e.g., phosphatidylcholine, triglycerides) that optimize the physicochemical properties of membranes for viral replication.

### 4.2. LDs as Functional Hubs in Flavivirus Infection

LDs serve as metabolic, structural, and signaling platforms during flavivirus infection (Figure 7).

(a) Metabolic roles:

LDs provide fatty acids and phospholipids required for the expansion of replication organelles. During DENV infection, lipolysis of LDs via adipose triglyceride lipase (ATGL) releases fatty acids that fuel β-oxidation and ATP generation, supporting the high energy demand of replication. Simultaneously, DGAT1-mediated re-esterification maintains LD turnover, preventing lipotoxicity. ZIKV infection exhibits a similar reliance on LD metabolism, but interestingly, ZIKV infection can lead to LD depletion in certain cell types (e.g., cardiomyocytes and neural progenitors), suggesting virus-specific remodeling of LD dynamics depending on host cell metabolic context [136,137].

(b) Structural roles:

LDs often physically associate with ER-derived replication compartments. Electron microscopy and confocal imaging reveal close apposition of LDs to viral RCs and envelope protein-enriched regions. Viral proteins such as DENV core (C) and NS4A/NS4B localize to LD surfaces, where they recruit replication or assembly complexes. The DENV C protein, in particular, anchors to LDs via hydrophobic and amphipathic helices, serving as a nucleation site for virion assembly. Inhibition of DGAT1, which mediates C protein targeting to LDs disrupts virion formation without affecting RNA replication, highlighting the structural importance of LD–ER contacts for assembly [138,139,140].

(c) Signaling roles:

LDs sequester or release signaling molecules that modulate antiviral responses. During infection, LD-resident proteins such as viperin, perilipin-3, and AUP1 participate in innate immunity and lipid homeostasis. Viperin, an interferon-stimulated gene (ISG), localizes to LDs and catalyzes the synthesis of ddhCTP, a nucleotide analog that inhibits viral RNA polymerases. Some flaviviruses counteract this defense by relocalizing viperin away from LDs or degrading it via proteasomal pathways, often regulated through UPR–ERAD crosstalk [141,142].

### 4.3. ER–LD Contact Sites: Structural Basis for Viral Replication and Stress Integration

ER–LD contact sites (MCSs) are emerging as key microdomains where lipid metabolism, proteostasis, and UPR signaling converge. These contacts are mediated by tethering complexes involving VAP-A/B, Rab18, DGAT2, and SNARE proteins (Figure 7). Through these junctions, LDs exchange lipids and proteins with the ER, contributing to membrane biogenesis and stress mitigation [26,143]. Flaviviruses appear to exploit these MCSs as replication hotspots. NS4A and NS4B, which induce ER curvature, also localize to MCS regions and may tether LDs to replication membranes. This spatial proximity facilitates lipid transfer, supplies membrane material, and potentially shields viral intermediates from cytosolic sensors [144,145].

Moreover, MCSs are enriched in UPR components such as BiP and XBP1s, suggesting a structural basis for the integration of lipid and stress responses. During DENV infection, VAP-A/B and Rab18 colocalize with replication complexes, and their knockdown reduces both LD association and viral replication [146]. ZIKV infection induces similar rearrangements but may also involve mitochondrial tethering, forming a tri-organelle network (ER–LD–mitochondria) that coordinates energy metabolism, redox balance, and stress signaling [51]. While UPR signaling promotes LD biogenesis, LDs reciprocally regulate UPR dynamics by buffering ER stress. LD formation sequesters excess lipids and misfolded proteins, reducing ER burden. Moreover, LDs can absorb lipotoxic intermediates and provide a sink for unfolded or aggregated proteins through ERAD-independent pathways. During recovery from ER stress, LDs serve as lipid donors for ER membrane repair and phospholipid replenishment. In the context of flavivirus infection, this feedback mechanism may prevent premature apoptosis and extend the lifespan of infected cells. However, excessive LD consumption (as observed during ZIKV-induced lipolysis or β-oxidation hyperactivation) can lead to metabolic exhaustion, mitochondrial stress, and cell death—pathological outcomes linked to tissue-specific damage such as ZIKV-induced microcephaly or cardiomyopathy.

Collectively, LDs act as both effectors and regulators of the UPR, forming a dynamic interface between lipid metabolism and ER proteostasis. Flaviviruses have evolved to exploit this interface by inducing UPR-driven lipogenesis, expanding ER–LD contacts, and co-opting LDs for replication and assembly. The interplay between the IRE1–XBP1, PERK–ATF4, and ATF6 pathways ensures continuous lipid supply and controlled stress adaptation, while LD feedback modulates ER stress by buffering excess lipids, supporting membrane remodeling, and fine-tuning UPR signaling to prevent proteotoxic collapse while sustaining viral replication.

## 5. Therapeutic Implications and Future Perspectives

The IRE1 pathway’s dual role of enhancing lipid synthesis while modulating immunity makes it a critical node exploited by flaviviruses. Inhibition of IRE1 RNase activity by small molecules such as 4μ8C or MKC8866 significantly reduces replication of DENV and ZIKV [70,147], suggesting therapeutic potential in targeting this axis. The intricate interplay among ER stress, UPR activation, and LD metabolism provides both opportunities and challenges for antiviral development. Flaviviruses exploit these host systems to ensure efficient replication, virion assembly, and persistence. As a result, pharmacological modulation of UPR pathways and lipid homeostasis represents a promising host-directed antiviral strategy. However, given the essential role of these pathways in normal physiology, therapeutic targeting must balance antiviral efficacy with cellular viability and systemic safety.

### 5.1. Targeting UPR Signaling as an Antiviral Strategy

Pharmacological inhibitors that modulate UPR pathways have demonstrated antiviral activity against flaviviruses. A PERK pathway inhibitor, such as GSK2606414, blocks eIF2α phosphorylation; alone, it has no effect on ZIKV replication but synergizes with the ATF6 inhibitor Ceapin-A7 to inhibit early and late infection [18]. The dengue virus inhibitor 4-HPR (fenretinide) suppresses DENV replication by inducing ATF4 activation independently of PKR-like ER kinase (PERK) and enhancing eIF2α phosphorylation in virus-infected cells [148]. These findings highlight the potential of combinatorial targeting across UPR branches to disrupt virus-induced stress responses while minimizing cytotoxicity.

### 5.2. Modulating Lipid Metabolism and LD Dynamics

Given the reliance of flaviviruses on lipid remodeling, inhibitors of lipid synthesis and LD formation have shown potent antiviral effects. DGAT1/2 inhibitors (e.g., A922500) suppress LD biogenesis, reducing viral replication and virion assembly in DENV- and ZIKV-infected cells [134,149]. AMPK activators, such as metformin and AICAR, downregulate lipogenesis and promote autophagy, thereby indirectly attenuating flavivirus replication [150]. In addition, PPAR agonists, by rebalancing lipid metabolism and inflammatory responses, have demonstrated partial antiviral activity in cell models [151]. Together, these data suggest that selective targeting of host lipid regulatory nodes offers a feasible strategy to block multiple stages of the viral life cycle.

### 5.3. Targeting Organelle Contact Sites and Host–Virus Interfaces

Emerging evidence indicates that ER–LD and ER–mitochondria contact sites are pivotal in flavivirus replication and metabolic adaptation [152]. Viral nonstructural proteins such as ZIKV NS4A/NS4B and HCV NS5A localize to membrane contact sites to orchestrate lipid trafficking and RC stabilization [69,84,153]. Interfering with host proteins mediating these contacts such as VAP-A/B, ACBD5, and Seipin may therefore impair viral replication [128]. Small molecules or peptides that disrupt these interfaces could selectively inhibit virus-induced membrane remodeling without broadly compromising cellular metabolism.

Another promising approach involves modulating ER-phagy and ER-associated degradation (ERAD) pathways. Compounds that enhance ER turnover or degrade viral proteins misfolded in the ER can restrict viral replication. For example, pharmacological activation of autophagy receptor FAM134B, which mediates selective ER degradation, has shown antiviral activity by limiting ER expansion during infection [154]. Similarly, enhancing p97/VCP-dependent ERAD can accelerate the clearance of viral components from the ER.

### 5.4. Challenges and Future Directions

Although targeting UPR and lipid metabolism pathways is mechanistically sound, translating these insights into safe antiviral therapies remains challenging. Most small molecules modulating UPR or lipid synthesis affect fundamental cellular functions, raising concerns about off-target toxicity and systemic lipid dysregulation. Therefore, target specificity, temporal control, and tissue selectivity will be critical considerations in future drug development.

A promising direction involves host-directed combination therapy, in which UPR modulators are used alongside direct-acting antivirals (DAAs) or immunomodulators to achieve synergistic effects. For example, combining IRE1 inhibitors with nucleoside analogs or interferon-based therapies may enhance antiviral potency while minimizing cytotoxicity. Moreover, the identification of virus-specific host dependencies, such as distinct lipid enzyme isoforms or contact site regulators selectively hijacked by flaviviruses, could guide the development of targeted therapeutics with minimal side effects.

Emerging technologies such as CRISPR-based functional genomics, single-cell transcriptomics, and lipidomics are revealing the fine-tuned regulatory networks connecting UPR signaling, lipid metabolism, and viral replication. Integration of these datasets will help define critical “nodes” for intervention. Furthermore, in vivo validation using animal models or human organoid systems is essential to assess the therapeutic feasibility of UPR–lipid axis targeting.

Finally, understanding virus-specific adaptations—for example, why ZIKV uniquely depletes LDs while DENV accumulates them—will provide mechanistic insight into the heterogeneity of virus–host interactions and may uncover shared vulnerabilities across flaviviruses and other positive-sense RNA viruses.

Flavivirus infection represents a delicate balancing act between viral exploitation and host defense within the ER–UPR–LD axis. The virus co-opts the UPR to expand membrane networks and redirect lipid resources, while the host activates stress responses to restore homeostasis or induce antiviral defenses. Deciphering this dynamic interplay has illuminated new facets of cell biology and revealed novel antiviral targets. As research advances, precise modulation of the UPR–LD network—guided by mechanistic understanding and systems-level analyses—holds great promise for the development of broad-spectrum, host-directed antiviral therapies against flaviviruses and related RNA pathogens.

## 6. Conclusions and Future Perspectives

The interplay between the UPR, LD metabolism, and flavivirus replication has emerged as a central theme in the understanding of virus–host interactions. The ER serves as both the origin and battlefield of this interaction, where flaviviruses remodel the membrane system to form RCs and exploit stress-adaptive signaling to sustain viral proliferation. In turn, the host cell activates the UPR through three major sensor pathways—PERK, IRE1, and ATF6—to restore homeostasis, regulate protein folding capacity, and reprogram lipid synthesis. The crosstalk among these adaptive responses determines whether the infection leads to productive replication, persistence, or cell death.

Mounting evidence shows that flaviviruses selectively harness UPR pathways to create a favorable intracellular environment. PERK signaling modulates translational arrest and antioxidant defense, while IRE1–XBP1 and ATF6 branches upregulate chaperones and lipid metabolic enzymes required for ER expansion and RC biogenesis. Viral nonstructural proteins, such as DENV NS4A/NS4B and ZIKV NS2B/3, manipulate these signaling cascades to reshape ER membranes and coordinate the recruitment of host lipid enzymes. Concurrently, flaviviruses reprogram lipid metabolism to generate and remodel LDs, which serve as lipid reservoirs, assembly platforms, or immune modulators. The UPR-driven transcriptional activation of SREBPs, DGATs, and ACC enhances the supply of phospholipids and triglycerides, enabling sustained membrane synthesis. This coupling between UPR activation and lipid remodeling not only supports viral replication but also alters cellular energy balance and innate immune signaling.

However, this virus–host relationship is not unidirectional. Prolonged or excessive UPR activation can shift the balance from adaptation to apoptosis, triggering host defenses through CHOP-mediated cell death, autophagy induction, and interferon signaling. In some contexts, such as severe dengue or ZIKV-induced neuropathogenesis, ER stress contributes to cytopathology and tissue injury. Understanding these dual outcomes—viral benefit versus host damage—is critical for deciphering pathogenesis and identifying therapeutic windows.

Future research must address several pressing questions. First, how do distinct flaviviruses fine-tune the temporal and spatial activation of each UPR branch? Second, what determines the differential utilization of LDs—accumulation in DENV versus depletion in ZIKV infection—and how does this affect RC formation and virion maturation? Third, what are the specific molecular mediators of ER–LD and ER–mitochondria contact sites that viruses hijack to coordinate lipid flux and energy metabolism? Answering these questions will require integrative approaches combining structural biology, lipidomics, proteomics, and single-cell transcriptomics to resolve dynamic virus–host interfaces in real time.

From a translational perspective, the UPR–LD network offers a wealth of potential antiviral targets. Host-directed strategies that modulate UPR signaling, lipid synthesis, or organelle contact sites could provide broad-spectrum efficacy against multiple flaviviruses. The development of selective PERK, IRE1, or ATF6 modulators, as well as inhibitors of lipid metabolic enzymes such as DGAT1/2 or SREBP regulators, represents an attractive direction. However, achieving specificity and minimizing toxicity will require a nuanced understanding of how these pathways function in different tissues and infection stages. Furthermore, combining UPR modulators with direct-acting antivirals or immune enhancers may yield synergistic outcomes.

In conclusion, the ER stress–UPR–LD axis represents a highly integrated cellular system that flaviviruses exploit to replicate, assemble, and persist. By illuminating the molecular choreography between viral factors and host stress responses, recent advances have redefined our view of cellular organelle biology during infection. Future work translating these mechanistic insights into therapeutic strategies holds great promise—not only for controlling flavivirus diseases but also for developing broad-spectrum antivirals targeting shared host vulnerabilities across diverse RNA viruses.

## Data Availability

No new data were created or analyzed in this study.
